# Randomized, double-blind, placebo-controlled, crossover trial of oral doxycycline for epistaxis in hereditary hemorrhagic telangiectasia

**DOI:** 10.1186/s13023-022-02539-8

**Published:** 2022-11-07

**Authors:** K. P. Thompson, J. Sykes, P. Chandakkar, P. Marambaud, N. T. Vozoris, D. A. Marchuk, M. E. Faughnan

**Affiliations:** 1 Toronto HHT Centre, St. Michael’s Hospital and Li Ka Shing Knowledge Institute, 30 Bond St, Toronto, ON M5B-1W8 Canada; 2 Division of Respirology, Department of Medicine, University of Toronto, Toronto, Canada; 3 The Feinstein Institutes for Medical Research, Northwell Health, Manhasset, NY USA; 4 Department of Molecular Genetics and Microbiology, Duke University Medical Center, Durham, NC USA; 5 Department of Respirology, St. Michael’s Hospital, Toronto, ON Canada; 6 Dalla Lana School of Public Health, University of Toronto, Toronto, ON Canada

**Keywords:** Hereditary hemorrhagic telangiectasia, HHT, Epistaxis, Biomarkers, Doxycycline, Angiogenesis

## Abstract

**Background:**

Vascular malformations in hereditary hemorrhagic telangiectasia (HHT) lead to chronic recurrent bleeding, hemorrhage, stroke, heart failure, and liver disease. There is great interest in identifying novel therapies for epistaxis in HHT given its associated morbidity and impact on quality of life. We aimed to measure the effectiveness of oral doxycycline for the treatment of epistaxis and explore mechanisms of action on angiogenic, inflammatory and pathway markers in HHT using a randomized controlled trial.

**Methods:**

13 HHT patients with epistaxis were recruited from the Toronto HHT Center at St. Michael’s Hospital. Recruitment was stopped early due to COVID-19-related limitations. The study duration was 24 months. Patients were randomly assigned to the treatment-first or placebo-first study arm. We compared the change in weekly epistaxis duration and frequency, biomarkers, blood measurements, and intravenous iron infusion and blood transfusion requirements between treatment and placebo.

**Results:**

There was no significant difference in the change in weekly epistaxis duration (*p* = 0.136) or frequency (*p* = 0.261) between treatment and placebo. There was no significant difference in the levels of MMP-9, VEGF, ANG-2, IL-6 or ENG with treatment. Hemoglobin levels were significantly higher (*p* = 0.0499) during treatment. Ferritin levels were not significantly different between treatment and placebo. There was no significant difference in RBC transfusions between treatment periods (*p* = 0.299).

**Conclusion:**

Overall, our study did not demonstrate effectiveness of doxycycline as a treatment for epistaxis in patients with HHT, though the study was underpowered. Secondary analyses provided new observations which may help guide future trials in HHT.

*Trial Registration* ClinicalTrials.gov, NCT03397004. Registered 11 January 2018 – Prospectively registered, https://clinicaltrials.gov/ct2/show/NCT03397004

**Supplementary Information:**

The online version contains supplementary material available at 10.1186/s13023-022-02539-8.

## Introduction

Hereditary hemorrhagic telangiectasia (HHT) is a rare autosomal dominant disease with an estimated prevalence of approximately 1 in 5000 [1]. It is characterized by vascular malformations of skin and mucous membranes of the nose and gastrointestinal tract, as well as the brain, liver and lung. Vascular malformations in HHT lead to chronic recurrent bleeding, as well as acute life-threatening hemorrhage, stroke, heart failure and liver disease. There are currently no medical therapies that have been proven by controlled trials to control the nearly universal epistaxis in HHT patients or to regress the VMs. International HHT Guidelines recommend moisturizing topical therapies, oral tranexamic acid and endoscopic ablative therapies as first and second line therapies [1]. For patients that fail to respond to these, systemic antiangiogenic therapies (such as intravenous bevacizumab) and surgical approaches are recommended [1]. As such, there is a growing interest in identifying medical therapies for HHT, in the hopes of reducing hemorrhage and its morbidity and mortality, with a particular interest in the development of therapies targeting regression or stabilization of vascular malformations.

Initial therapeutics interest has been in antiangiogenic therapies, including anti-VEGF therapies, such as bevacizumab and pazopanib. Several uncontrolled series have reported that intravenous (IV) bevacizumab reduced epistaxis, improved anemia, reduced transfusion requirements, or improved quality of life (QOL) [2–12]. Small studies of pazopanib have also reported reduced epistaxis, improved hemoglobin, reduced transfusion and iron requirements, and improved QOL scores [13, 14], however there have been no controlled trials of systemic antiangiogenic therapies in HHT reported to date. In addition, small series of intranasal injected bevacizumab have shown only modest and brief benefits [15, 16], while recent multicenter randomized controlled trials (RCTs) of topical therapies have been negative [17, 18]. Interestingly, there has been no convincing evidence of macroscopic regression of VMs in IV bevacizumab series, even in cases with marked reduction in bleeding. Though there are ongoing trials of systemic therapies investigating benefits and safety in HHT, highly-effective long-term therapies for chronic bleeding in HHT have yet to be proven [1]. In addition, many agents currently being investigated, including bevacizumab and pazopanib are costly, often associated with significant toxicity, administered intravenously and may only be appropriate for patients with more severe disease. There remains, therefore, a pressing need to identify effective agents for the full range of disease severity in HHT, and with the potential to regress or stabilize vascular malformations.

This study investigates doxycycline, given its demonstrated anti-angiogenic and anti-inflammatory properties, as well as compelling effects in arteriovenous malformation (AVM) models. Doxycycline suppresses VEGF-induced cerebral MMP-9 activity in vivo in the mouse model [19], and has anti-inflammatory effects as well, via inhibition of pro-inflammatory cytokines [20]. In human brain VM tissue, there is evidence of increased expression of MMP-9 [21] and VEGF [22] and another tetracycline, minocycline, has attenuated brain hemorrhage in the mouse [23]. Recently, a small retrospective case series reported sustained reduction in epistaxis in seven HHT patients treated with oral doxycycline [24], though a very recent clinical trial (performed during the same period as our trial) of 2 months of doxycycline in HHT patients showed no benefit [25]. Doxycycline also has the advantages of a proven safety track record for long-term use, oral administration and low cost.

We aimed to investigate the effectiveness of oral doxycycline for the treatment of recurrent epistaxis in HHT and explore potential mechanisms of action of doxycycline on inflammatory, angiogenic, and BMP9-ALK1-Smad1/5/9 pathway markers.

## Methods

*Design and Recruitment* Phase II, double-blind, randomized, placebo-controlled, crossover trial of oral doxycycline in HHT subjects with moderate-severe recurrent epistaxis. 13 HHT patients with moderate to severe epistaxis were recruited between August 2018 and December 2019 from the Toronto HHT Center at St. Michael’s Hospital. Study inclusion and exclusion criteria are summarized in Table 1. The study was approved by the Research Ethics Board at St. Michael’s Hospital (REB) and was funded by the US Department of Defense.

**Table 1:** Inclusion and exclusion criteria

Inclusion criteria	Exclusion criteria
Age > = 18 Clinical HHT diagnosis or genetic diagnosis of HHT Epistaxis at least 15 min per week (mean for past month) At least one telangiectasia (skin or mucosal) available for micro-imaging Ability to give written informed consent	Allergy/intolerance to the study drug or related agents Unstable medical illness Acute infection Creatinine > ULN (upper limit of normal) Liver transaminases (AST or ALT) > = 2 × ULN Recent (within 2 months) use of study drug or other tetracycline agents Women who are pregnant or breastfeeding or plan to become pregnant during the study BHCG level>6IU/L (re-test if 6-24 IU/L) Specific contra-indications for study drug On blood thinner AND refuses to have family doctor notified of study participation

**Fig. 1:**
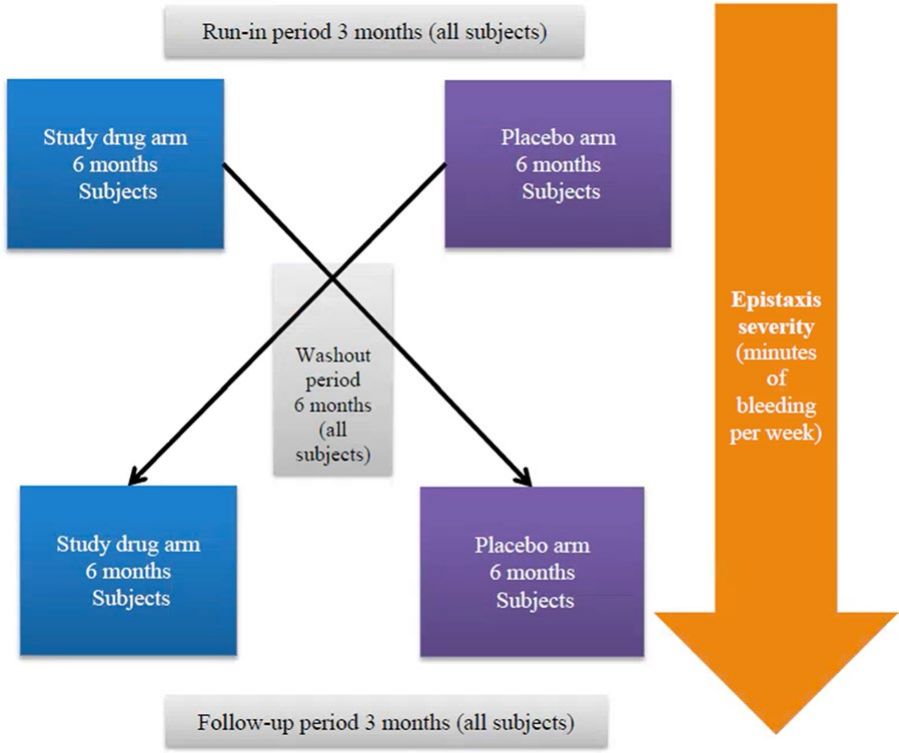
Study design

The duration of the study was 24 months and consisted of a run-in period of 3 months, a treatment period of 6 months (study drug/placebo), a washout period of 6 months, a second treatment period of 6 months (study drug/placebo) and finally a follow-up period of 3 months (Fig. 1). During the two investigational product periods, subjects were instructed to orally administer 100 mg of doxycycline hyclate or placebo two times daily, every 10–12 h. The specific dosage of doxycycline, at 100 mg twice daily was based on previous clinical experience [24]. The 3-month run-in period was designed to ensure accurate baseline measurement of epistaxis. The 6-month washout period between investigational products was used to allow for symptom return to baseline (previous studies, such as the NOSE trial, have used 3-month washout periods, which were observed to be insufficient [18]), and for drug and placebo arms to occur over the same seasons [26, 27], for each participant. Patients continued with their routine clinical care during the study. None underwent surgical or endoscopic treatments for epistaxis during the study period. The 3-month follow-up period was used to obtain both safety and outcome data. Exit interviews were conducted with each participant during their final study visit.

We estimated recruiting 22 participants would give us 80% power to detect a 20% difference in weekly total epistaxis (arbitrarily defined), assuming an untreated baseline of approximately 30 min and an estimated standard deviation of 10 min, at a two-sided significance level of 0.05. We planned for 30 participants for the trial, to be conservative and to allow for attrition/drop-out. We stopped trial recruitment early due to COVID-19-related recruitment limitations.

All subjects underwent a screening visit for eligibility review and informed consent. The order of investigational product was randomized according to a pre-determined, computerized block randomization scheme using randomly permuted block sizes. Randomization was performed by the research pharmacy staff; participants, other study personnel, the treating physician, and other outcome assessors were blinded. During the two investigational product periods, subjects were instructed to orally administer 100 mg of doxycycline hyclate or placebo two times daily, every 10–12 h.

*Data Collection* Patients were asked to keep a daily diary of all epistaxis events, recording duration and intensity, throughout the entire study period. Patient daily diaries were collected by the research team every 2 weeks. Completion rate was calculated as the total number of days with complete data over the total number of days.

Safety and adverse event monitoring occurred every 2 weeks by phone and every 6 weeks at each clinic visit during the two investigational product periods. Study participants were monitored for typical side effects of the study drug, including symptoms of photosensitivity, vertigo, candidiasis, and gastrointestinal intolerance. Additionally, participants were monitored for the following adverse events; hypertension, infection, change in blood pressure, change in electrolytes, renal function, liver function, routine hematology or drug levels, and pregnancy in women.

Physical examination, epistaxis severity score (ESS) assessment, and blood measurements related to chronic bleeding, including hemoglobin and ferritin were performed at each clinic visit. IV iron infusion data (including the date and amount of iron) and red blood cell (RBC) transfusions (date and number of units) were also collected.

Serum and plasma samples were collected every 12 weeks and measured for inflammatory, angiogenic, and BMP9-ALK1-endoglin-Smad1/5/9 pathway markers (VEGF, MMP-9, endoglin (ENG), ANG-2 and IL-6), as per manufacturer instructions (Quantikine ELISA kits, R&D Systems).

*Primary Outcome* The change in weekly epistaxis duration (WED) between treatment and placebo, compared to baseline.

*Secondary outcomes* Changes in epistaxis frequency and severity, blood hemoglobin and ferritin, serum and plasma biomarkers, and IV iron and RBC transfusion requirements between treatment and placebo.

### Primary statistical analysis

Continuous variables were summarized descriptively as median and range. Categorical variables were summarized as frequency and proportion. A weekly total of epistaxis bleeding (minutes per week) was calculated from a sum of duration of all bleeding events each week, as measured from daily patient diaries. Mean WED at treatment baseline (weekly totals averaged over the last 4 weeks before treatment) was compared with mean WED averaged over the last 4 weeks of treatment. Similarly, mean WED averaged over the last 4 weeks before placebo was compared with mean WED averaged over the last 4 weeks of placebo. Finally, the change in mean WED on treatment was compared to the change in mean WED on placebo, and reported as the primary outcome.

All analyses were conducted using the open source software R version 4.0.3. All *p*-values are two-sided and assessed at < 0.05, unless otherwise stated.

### Secondary outcomes statistical analyses

The primary statistical method was also used to calculate the change in mean weekly epistaxis frequency (number of nosebleeds) between treatment and placebo.

Biomarker measures (duplicate measures) were first compared between samples collected at the start and end of treatment. Biomarker measures were then compared between samples collected at the start of placebo and the end of placebo. The change on treatment was compared to the change on placebo for each biomarker, respectively. For patients with missing biomarker measurements, the measurement obtained from the subsequent sample collection was used, when available, to reduce exclusion of individual patients from the analysis. Close-to-zero or negative values indicated non-detectable levels of biomarker and were corrected to 1 ng/mL for the purpose of the analysis.

Mean hemoglobin and ferritin levels during the treatment and placebo periods were calculated from blood measurements obtained at each clinic visit and compared. Blood measurements collected during the first week of the treatment and placebo periods (week 12 and week 60) were excluded from the analysis, as to account for subjects starting drug at different times of the week.

## Results

### Study participants

Thirteen patients were recruited between September 2018 and October 2020, of whom, 6 (46.2%) were male. The mean age at study entry was 62.7 ± 13.8 (range 37.0–89.6). Seven participants were randomized to the treatment-first arm (Allocation A), and six to the placebo-first arm (Allocation B). One participant in the treatment-first arm withdrew from the study early due to COVID-19 pandemic stress, while a second participant was withdrawn from the study after reporting blurred vision, as associated intracranial hypertension could not be ruled out. A third participant discontinued study tablets during the second treatment period (which was later determined to be the placebo period) due to unrelated medical issues but remained in the study for its entirety. Doxycycline was well-tolerated, with zero serious adverse events (SAE’s) observed.

Eleven participants completed the study, 5 in the treatment-first arm (Allocation A) and 6 in the placebo-first arm (Allocation B). Baseline characteristics are presented in Table 2. There were no significant differences in baseline characteristics between Allocation groups. Allocation A consisted mostly of males (80.0%), while Allocation B consisted mostly of females (66.7%). Allocation B had a higher median ESS at enrollment (5.69 vs 4.17), however median ESS at randomization was similar between the two groups (4.60 and 4.35, respectively). Within the Allocation B group, 50% received at least one IV iron infusion during the 3-month run-in, with a mean WED of 47.7 min. In contrast, 0% of patients in the Allocation A group received IV iron infusions during the run-in period, and the mean WED was only 28.7 min. The completion rate of the daily epistaxis diary was 98.8% for the eleven participants.

**Table 2:** Baseline characteristics

Characteristic	Allocation A (*n* = 5)*	Allocation B (*n* = 6)*	Overall (*n* = 11)*
Age	67.4 (60.7–89.6)	64.3 (42.5–70.7)	66.2 (42.5–89.6)
Male	4 (80.0%)	2 (33.3%)	6 (54.5%)
Mutation			
ACVRL1	2 (40.0%)	5 (83.3%)	7 (63.6%)
Endoglin	3 (60.0%)^a^	1 (16.7%)	4 (36.4%)
Brain VM	1 (20.0%)	0 (0.0%)	1 (9.1%)
Pulmonary AVM	1 (9.1%)	1 (16.7%)	3 (27.3%)
Symptomatic liver VM	1 (20.0%)	2 (33.3%)	3 (27.3%)
GI bleeding	4 (80.0%)	2 (33.3%)	6 (54.5%)
Anemia (at enrollment and/or randomization)	4 (80.0%)	4 (66.7%)	8 (72.7%)
Enrollment ESS	4.17 (2.43–5.43)	5.69 (4.42–7.28)	4.96 (2.43–7.28)
Randomization ESS	4.35 (3.44–4.53)	4.60 (2.62–7.28)	4.35 (2.62–7.28)
IV iron infusions (during run-in)	0 (0.0%)	3 (50.0%)	3 (27.3%)
Mean infusion amount (mg) (during run-in)	NA	300 (125–300)	300 (125–300)
Blood transfusions (during run-in)	0 (0.0%)	0 (0.0%)	0 (0.0%)
Enrollment Hgb	129 (111–148)	121 (101–128)	123 (101–148)
Randomization Hgb	133 (113–159)	117 (100–132)	126 (100–159)
Mean weekly epistaxis duration	28.7 (21.3–274.4)	47.7 (17.8–142.1)	34.3 (17.8–274.4)

*Values are median (range) or *n* (%) at enrollment, unless otherwise stated

^a^1 patient had a variant of unknown significance in the Endoglin gene

### Primary outcome

**Fig. 2:**
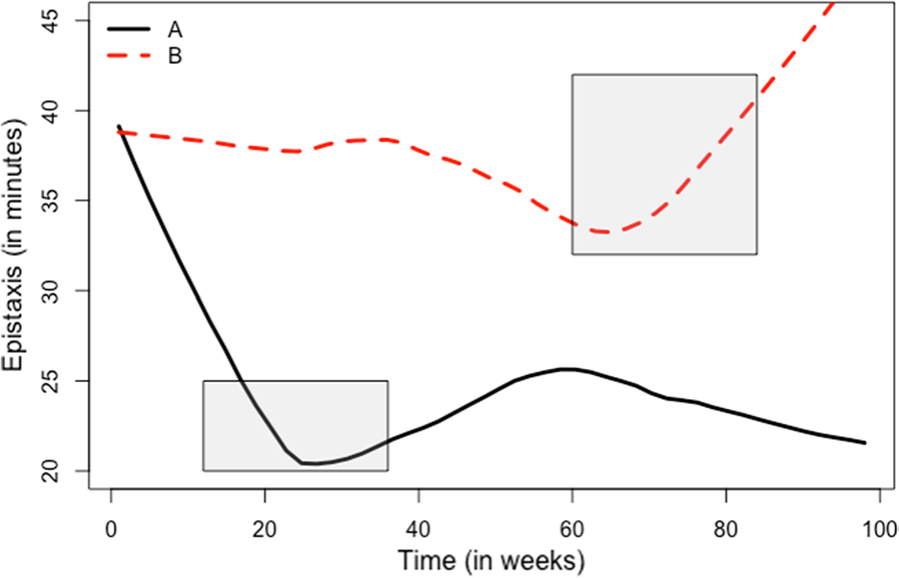
Weekly epistaxis duration by treatment allocation. Shaded boxes represent periods of doxycycline treatment

There was no significant difference between the change in WED on treatment and placebo (*p* = 0.136). Figure 2 depicts a smooth line plot of WED, by treatment allocation. The mean WED was 48.1 min (SD = 58.9) at treatment baseline and 48.8 min (SD = 46.8) at the end of treatment. The mean WED at placebo baseline was 61.1 min (SD = 55.3). At the end of placebo, the mean WED was 47.2 min (SD = 55.3). On average, the change in WED on treatment was actually 0.41 min higher than on placebo. Patients showed an average increase of 0.29 weekly minutes of epistaxis on treatment and an average decrease of 0.11 weekly minutes of epistaxis on placebo. Overall, the change in WED between treatment and placebo was neither clinically nor statistically significant.

### Secondary outcomes

**Fig. 3:**
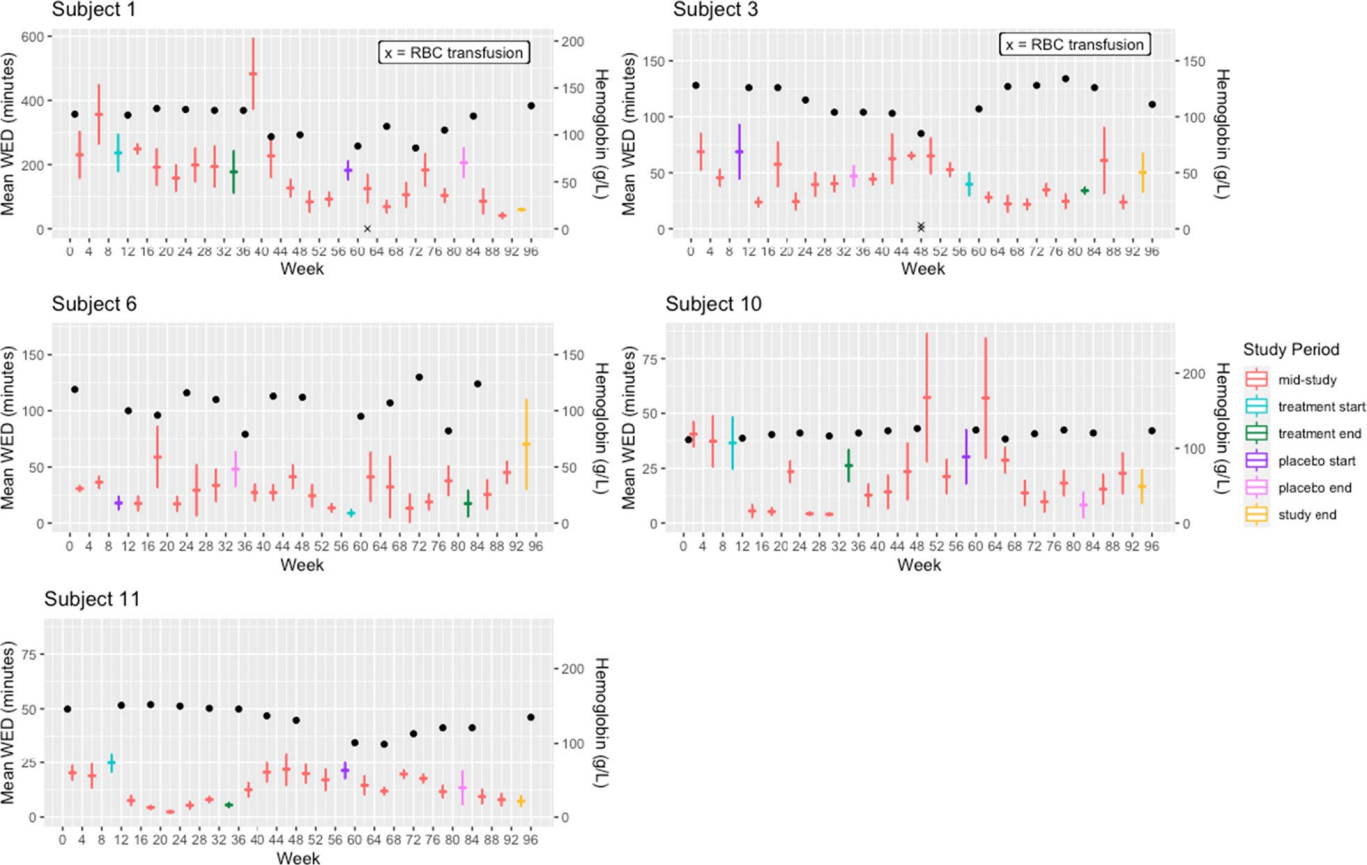
Mean WED, hemoglobin level and RBC transfusions for individual patient responders. Horizontal tick marks represent mean WED for that month and error bars represent standard deviation of the mean. Hemoglobin levels are depicted by black circles and blood transfusions are indicated by an x, with each x representing 1 unit. In general, patients in Allocation B had more severe epistaxis than patients in Allocation A. The scale for mean WED was set to 87 min for Allocation A patients, except for Subject 1, who was a major outlier, with a maximum of 483 min. The scale for mean WED was set to 150 min for 5/6 Allocation B patients and 250 min for the remaining 1 patient

**Fig. 4:**
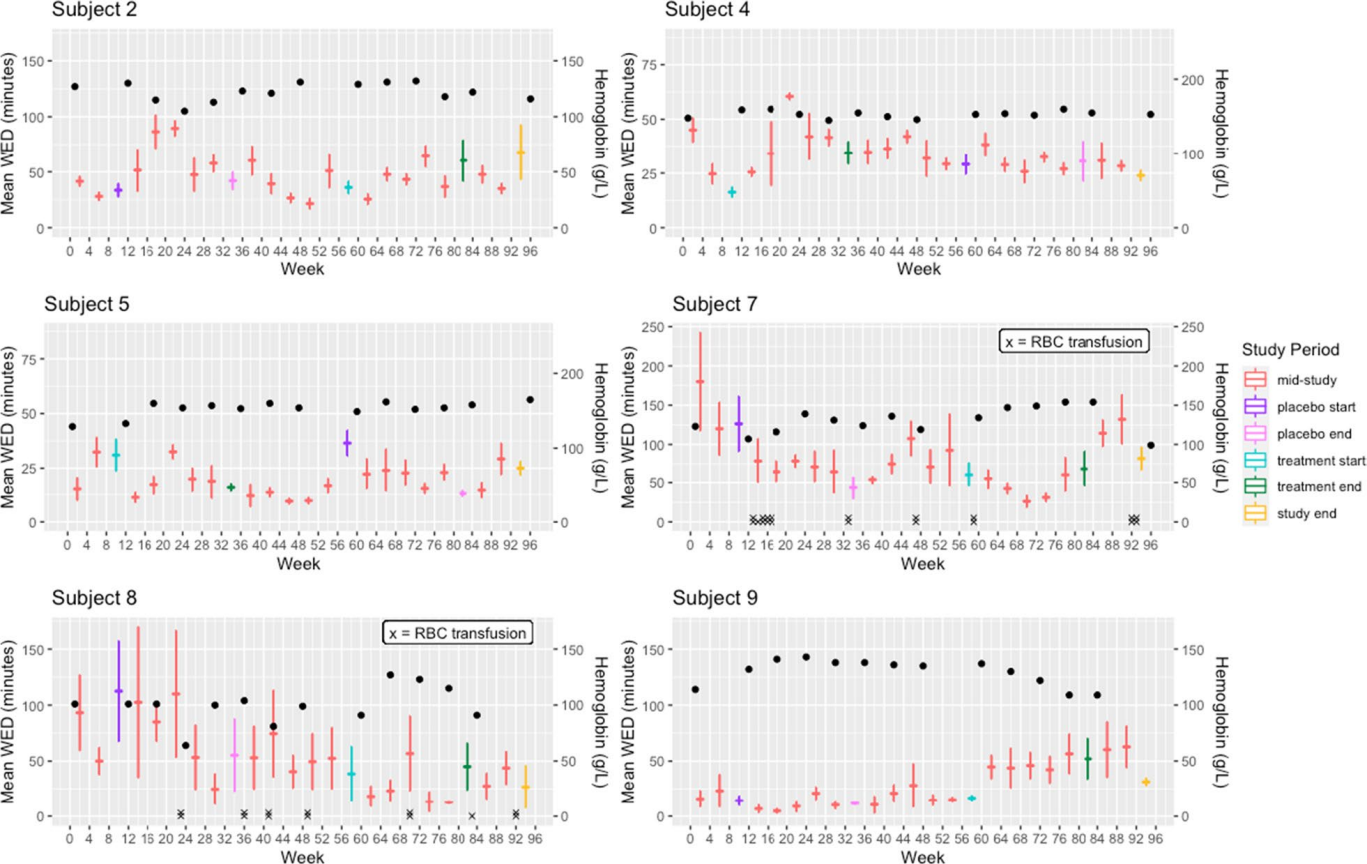
Mean WED, hemoglobin level and RBC transfusions for individual patient non-responders. Horizontal tick marks represent mean WED for that month and error bars represent standard deviation of the mean. Hemoglobin levels are depicted by black circles and blood transfusions are indicated by an x, with each x representing 1 unit. In general, patients in Allocation B had more severe epistaxis than patients in Allocation A. The scale for mean WED was set to 87 min for Allocation A patients, except for Subject 1, who was a major outlier, with a maximum of 483 min. The scale for mean WED was set to 150 min for 5/6 Allocation B patients and 250 min for the remaining 1 patient

There was no significant difference between the change in weekly epistaxis frequency on treatment and placebo (*p* = 0.261). The mean weekly epistaxis frequency at treatment baseline was 7.5 nosebleeds (SD = 3.6). The mean weekly epistaxis frequency at the end of treatment was 6.8 nosebleeds (SD = 3.0). The mean weekly epistaxis frequency was 8.9 (SD = 4.1) at placebo baseline and 6.2 (SD = 3.3) at the end of placebo. The average change in weekly number of nosebleeds on treatment was 0.24 greater than on placebo. Patients showed a slight, non-significant increase in weekly number of nosebleeds on treatment (0.02) and a slight, non-significant decrease in weekly number of nosebleeds on placebo (0.22). Secondary post-hoc analyses of mean WED plots (Figs. 3 and 4), categorizing patients as Responders vs. Non-Responders, are detailed in Addititional file 1.

There was no significant difference in the levels of MMP-9, VEGF, ANG-2, IL-6 or ENG between treatment and placebo, even when stratified by treatment allocation group.

Across the group, hemoglobin levels were significantly higher (mean difference = 11.14, *p* = 0.0499) during the treatment period, compared to the placebo period. There was no significant difference between ferritin on treatment versus placebo.

A total of 5 (45.5%) patients received IV iron infusions during the study period; 2 in Allocation A and 3 in Allocation B. Three patients received a higher number of iron infusions during the placebo period, 1 patient received the same number of infusions during both periods, and the last patient received only one more infusion during treatment, compared to placebo (6 vs 5). In addition, 4 of these patients (36.4%, overall) received at least one RBC transfusion during the study (1 Allocation A patient and 3 Allocation B patients). There was no significant difference in RBC transfusions between treatment and placebo periods (*p* = 0.299). (refer to Figs. 3 and 4).

### Exit interviews

Exit interviews were conducted with each participant during their final study visit. Participants were asked a series of questions regarding perceived benefit of the trial/drug, order of investigational product received, and attitudes toward continuing treatment with doxycycline in the future. 9/11 (81.2%) participants reported a benefit from being in the trial. The remaining 2 participants reported no benefit or were unsure of any benefit. The most frequently reported benefits included less nosebleeds (6), less severe nosebleeds (2), improved hemoglobin and/or ferritin (3), and fewer blood transfusions (1). Next, 9/11 (81.2%) participants correctly identified the order of investigational product by the end of the study. In addition, 10/11 (90.9%) participants reported that they would like to take doxycycline after the trial ends.

## Discussion

We observed no significant difference in the change in WED between treatment and placebo, suggesting oral doxycycline may not be effective for the treatment of epistaxis in HHT patients, though the study was underpowered. However, several interesting secondary observations of individual patient responses described here may help guide future trials in HHT.

Rare disease presents a number of challenges in clinical trial design, including recruitment challenges, related power limitations and less knowledge about outcomes measurement [28]. Considering these limitations, as well as the large variability in epistaxis measures across HHT patients, a crossover-trial design, with each subject receiving the study drug and placebo, and therefore serving as their own control, was planned, to limit bias in measuring this subjective outcome. The 6-month washout period also allowed for drug and placebo arms to occur during the same season, for each participant, to minimize the effect of seasonal epistaxis variation [26], which has been previously documented in HHT [27]. Despite this relatively long washout period, we did not observe a decrease in patient enthusiasm/compliance or study attrition during the trial. Unfortunately, we stopped trial recruitment earlier than planned, at approximately half the proposed sample size, due to recruitment restrictions during COVID times, underpowering our study. Our observations to date suggest that oral doxycycline may not be an effective drug for the treatment of epistaxis in most patients with HHT, and are in agreement with another recent clinical trial of 22 patients [25]. We confirmed that WED collection by daily diary was feasible, with an excellent completion rate of 98.8%. However, observed variability of the measure reinforces the need for larger sample size, and will provide crucial data for planning for future trials in HHT. In addition, we observed a broad range of WED across study participants, and this is an additional variable that may need to be considered in future studies.

We found that hemoglobin concentration was significantly higher among patients during the treatment period of the study, as compared to the placebo period. Anemia secondary to iron deficiency from recurrent epistaxis and/or GI bleeding is common in HHT, with an estimated prevalence up to 50% [29]. Affected patients require chronic oral and/or IV iron replacement therapy and, in severe cases, RBCs. Moreover, anemia is commonly associated with weakness, fatigue, decreased exercise tolerance, headache, irritability, and poor quality of life [30, 31]. Importantly, IV iron infusions and RBC transfusions were not responsible for the elevation in hemoglobin reported here, as most patients actually received fewer infusions and transfusions during the treatment period.

We found no significant difference in the levels of inflammatory, angiogenic and BMP9-ALK1-Smad1/5/9 pathway markers between the doxycycline and placebo periods. Doxycycline has previously demonstrated anti-angiogenic and anti-inflammatory properties, including through the suppression of cerebral MMP9 activity [19], and the inhibition of pro-inflammatory cytokines and chemokines [20]. Despite evidence of increased expression of these key biomarkers in human vascular malformation tissue [21, 22], our findings do not suggest angiogenic, inflammatory or pathway effects of doxycycline. However, responders to doxycycline had a significantly higher baseline ANG-2 level compared to non-responders. ANG-2 plays an important role in diseases related to vascular permeability and angiogenesis. Its role has been primarily explored in tumor-induced angiogenesis, where its inhibition or overexpression decreased or increased tumor size and metastatic efficacy, respectively [32–34]. In recent years, the angiogenic activity of ANG-2 has been identified as context-dependent [35]. One such context is the expression of other angiogenic factors, such as VEGF, as ANG-2 is found to induce permeability and angiogenesis in the presence of VEGF but lead to vessel regression and endothelial cell death in its absence [36]. In HHT, anti-VEGF treatment has been found to restore tissue TSP-1 and ANG-2 levels and improve the microvascular phenotype [37], as well as prevent the formation of AVMs in mouse models [38]. ANG-2 is transcriptionally upregulated in several HHT mouse models, and its neutralization improves AVM pathology [39, 40], suggesting that ANG-2 cross talks with VEGF during the pathogenic process of HHT to promote angiogenesis. ANG-2 is also important for the initiation of the inflammatory response, as ANG-2-deficient mice are unable to initiate an inflammatory response in short-term infection models, and treatment with recombinant ANG-2 is found to restore the inflammatory response [41]. Moreover, serum ANG-2 levels have been positively associated with inflammatory biomarkers, including C-reactive protein and white blood cell count [42]. Further, ELISAs from this study show a correlation between ANG-2 and IL-6 levels, across all patients and treatment conditions, strengthening the association between ANG-2 elevation and inflammation. It is therefore possible that the higher baseline ANG-2 levels among responders may have promoted anti-angiogenic activity in the absence of VEGF and/or an increased ability to initiate an inflammatory response, leading to better outcomes. Responders to doxycycline also had a near-significantly higher baseline IL-6 level compared to non-responders. IL-6 is known to be a pro-inflammatory cytokine involved in inflammation, immune response, and hematopoiesis. An increased susceptibility to infections has been observed in mice with HHT mutations, possibly due to defects in phagocytic activity, alterations in leukocyte recruitment, and a reduction in proinflammatory cytokines, including IL-6 [43]. However, IL-6 has also been found to enhance anti-angiogenic therapy in certain diseases, such as ovarian cancer, by suppressing ANG-1 expression and promoting dependence on VEGF for angiogenesis [44]. Thus, the higher IL6 levels among responders at baseline may have supported the anti-angiogenic properties of doxycycline, leading to an improvement in epistaxis. Our observations suggest there may be different patient phenotypes identifiable with biomarkers, which may influence response to doxycycline or other therapies.

### Limitations

Our study has a few limitations. First, the study was underpowered. However, we believe the findings presented here offer valuable insight into HHT treatment response heterogeneity and important lessons for future studies of epistaxis treatment in HHT. Second, though this was a crossover trial, with each subject receiving the study drug and placebo, and therefore serving as their own control, our analysis of severe nosebleeds indicated a significant difference in nosebleed severity between the two treatment groups that suggests suboptimal results of randomization. In the case of our negative trial, this difference could have led to us missing an effect. Patients should potentially be stratified based on nosebleed variability and severity in future RCTs in HHT. Further, the majority of participants correctly identified their study arm. Although this may point to an “unseen” benefit of doxycycline that requires further investigation, it also suggests ineffective blinding of study participants. Though this likely did not influence our results, as it was a negative trial, effective blinding in future similar trials will be essential to minimize bias and maximize the validity of the results. Further research into factors that enable correct identification of the study arm by participants, as well as strategies to ensure effective blinding in the future should be a priority. Finally, although WED may be indicative of severity, it may not provide a comprehensive picture, in that patients with frequent shorter bleeds and less frequent longer bleeds would have similar WED, but may be differently affected. Therefore, correlating WED with quality of life measurement tools, or including a measure of severe nosebleeds in the future may be beneficial.

## Conclusion

Oral doxycycline did not reduce mean WED or frequency, in this underpowered trial, suggesting it may not be effective for the treatment of epistaxis in HHT. There was no indication of inflammatory, angiogenic, or pathway effect of treatment with doxycycline. On secondary post-hoc analyses, qualitative responders were characterized by a history of chronic GI bleeding and anemia, IV iron and RBC transfusion dependence, and higher baseline ANG-2 levels. Overall, two prospective clinical trials of oral doxycycline have now been completed and have both been negative, suggesting that doxycycline has limited use in HHT (Additional file 1).

## Supplementary Information

**Additional file 1**. On-line Supplementary Materials.

## Data Availability

The datasets generated during and/or analyzed during the current study are not publicly available due to them containing information that could compromise research participant privacy/consent but are available from the corresponding author on reasonable request.
