# Supplementary material for: Randomized, double-blind, placebo-controlled, crossover trial of oral doxycycline for epistaxis in hereditary hemorrhagic telangiectasia
Source: Orphanet J Rare Dis. 2022 Nov 7;17:405. doi: 10.1186/s13023-022-02539-8 (PMC9640829; doi:10.1186/s13023-022-02539-8)
Supplement: Supplementary file 1 — Additional file 1. On-line Supplementary Materials. [file 13023_2022_2539_MOESM1_ESM.docx]

*Secondary Post-hoc Analyses: Responders vs. Non-Responders*

Patients were categorized as “responders” or “non-responders” based on qualitative review of mean WED plots (**Fig. 3** and **Fig. 4**). Responders included patients who demonstrated a negative change in mean WED on treatment, or a lesser positive change in mean WED on treatment, compared to placebo. Non-responders included patients who demonstrated no change, or a greater positive change in mean WED on treatment than placebo. The changes in biomarker levels between treatment and placebo were recalculated with stratification based on qualitative response. Baseline biomarker measures, collected at week 12, were also compared between responders and non-responders.

*Secondary Post-hoc Analyses: Responders vs. Non-Responders:*

Based on qualitative observation, 5 patients (subjects 1, 3, 6, 10, and 11) were categorized as “responders” (details below and Fig. 3) and 6 patients (subjects 2, 4, 5, 7, 8, and 9) were categorized as “non-responders” (details below and Fig. 4). There was no association between treatment allocation and observed response, with 3 responders and 2 non-responders in Allocation A, and 2 responders and 4 non-responders in Allocation B. Interestingly, though, 4/5 (80%) responders had known chronic GI bleeding, compared to only 2/6 (33.3%) non-responders. Further, 3/5 (60%) responders received IV iron infusions during the study, compared to only 2/6 (33.3%) non-responders. Similarly, 2/5 (40%) responders received RBC transfusions, as opposed to 2/6 (33.3%) non-responders. There were also 4 (80%) responders with anemia, compared to 4 (66.6%) non-responders.

There was no significant difference in the levels of MMP-9, VEGF, ANG-2, IL-6 or ENG between treatment and placebo, even when stratified by responders and non-responders. There was, however, a significant difference (p=0.037) in baseline ANG-2 levels between responders and non-responders. Responders had an average ANG-2 baseline of 5361.00 pg/mL, while non-responders had a significantly lower average baseline of 2973.42 pg/mL. There was also a borderline significant difference (p=0.062) between baseline IL6 levels, with an average of 23.49 pg/mL for responders and 21.34 pg/mL for non-responders. There was no significant difference in baseline levels of MMP9 (p=0.126), VEGF (p=0.182), or ENG between responders and non-responders (p=0.771).

*Responders*

Responders included subjects 1, 3, 6, 10 and 11 **(Fig. 3)**.

Subject 1 had the most severe epistaxis of all participants in the trial, with WED decreasing on treatment and increasing on placebo. Hemoglobin was consistently higher during the treatment period, and more variable during the placebo period. Further, this patient’s only blood transfusion occurred during the placebo period. Interestingly, there was a decreasing trend in WED throughout the 24-month study period, after receiving doxycycline in the first treatment period.

Subject 3 experienced a decrease in WED during both treatment and placebo periods. Importantly though, the doxycycline period was characterized by shorter WED and higher hemoglobin levels. This patient also exhibited a distinctive, symmetrical pattern in mean WED that was closely reflected by hemoglobin level, suggesting a seasonal effect on WED, despite the 6-month washout period.

Subject 6 had less of an increase in weekly epistaxis on treatment, compared to placebo, suggesting some level of response. In general, though, WED and hemoglobin levels were quite variable over the 24-month study period.

Subject 10 experienced a decrease in mean WED during both treatment and placebo. Notably though, the shortest WEDs all occurred during the treatment period. Additionally, there was much greater variability in epistaxis duration during the washout and placebo periods, with two months of particularly high and variable epistaxis evident on the plot. Hemoglobin levels were fairly consistent throughout the study.

Subject 11 had a decrease in mean WED from the start to end of doxycycline treatment, as well as short epistaxis durations with minimal variability throughout the treatment period. Hemoglobin levels were highest when WED was lowest.

*Non-Responders*

Non-responders included subjects 2, 4, 5, 7, 8 and 9 **(Fig. 4)**.

There was no discernable effect of doxycycline on subject 2, who showed an increase in WED during both treatment and placebo, as well as a high degree of variability within each period. Hemoglobin levels were higher when WED was lower.

Subject 4 exhibited a notable increase in mean WED on treatment and no change in mean WED on placebo. Hemoglobin levels were relatively consistent throughout the study.

Participant 5 did not appear to respond to doxycycline, with a similar change in WED observed during both treatment and placebo. The least amount of epistaxis occurred during the washout period. Hemoglobin levels were lowest during the run-in period, when WED was higher.

Subject 7 demonstrated a sharp decrease in mean WED on placebo and a slight increase in mean WED on treatment. Importantly though, treatment with doxycycline appeared to be associated with higher hemoglobin levels, fewer blood transfusions, as well as the two study months with the least amount of epistaxis.

Similarly, subject 8 demonstrated a decrease in epistaxis duration on placebo and a slight increase in epistaxis duration on treatment. However, the mean WEDs of the intervening months were lower during the treatment period, compared to the placebo period. Furthermore, the treatment period appeared to be associated with less epistaxis variability and higher hemoglobin levels.

Subject 9 demonstrated a negative response to doxycycline. The treatment period was associated with increased epistaxis duration, greater variability, as well as an overall increase in mean WED, as well as a decreasing trend in hemoglobin.
